# A Treatise on Practical Chemistry, Etc.

**Published:** 1885-08

**Authors:** 


A Treatise on Practical Chemistry and Qualitative Inorganic Analysis. *By* Frank Clowes, d. sc. *Philadelphia:* Lee Bros. & Co.; *Chicago,* Jansen, McClurg & Co.

This work, which now appears in the third American edition, is intended for general laboratory students, and is one of the best of its kind in use.

The first part of the book deals with the preparation and properties of various simple substances, solids, liquids and gases, while following sections take up, systematically, the reactions and processes of qualitative analysis.

It is well written, clear and full enough for the general student, while for those making a specialty of chemistry it serves as a good introduction. J. H. L.

